# Interaction between the human papillomavirus 16 E7 oncoprotein and gelsolin ignites cancer cell motility and invasiveness

**DOI:** 10.18632/oncotarget.8646

**Published:** 2016-04-08

**Authors:** Paola Matarrese, Claudia Abbruzzese, Anna Maria Mileo, Rosa Vona, Barbara Ascione, Paolo Visca, Francesca Rollo, Maria Benevolo, Walter Malorni, Marco G. Paggi

**Affiliations:** ^1^ Department of Therapeutic Research and Medicines Evaluation, Istituto Superiore di Sanità, Rome, Italy; ^2^ Department of Research, Advanced Diagnostics and Technological Innovation, Unit of Cellular Networks and Therapeutic Targets, Regina Elena National Cancer Institute, IRCCS, Rome, Italy; ^3^ Department of Research, Advanced Diagnostics and Technological Innovation, Unit of Immunology and Immunotherapy, Regina Elena National Cancer Institute, IRCCS, Rome, Italy; ^4^ Unit of Pathology, Regina Elena National Cancer Institute, IRCCS, Rome, Italy; ^5^ Istituto San Raffaele Pisana, Rome, Italy

**Keywords:** human papillomavirus 16 E7, gelsolin, actin, cancer, cell migration

## Abstract

The viral oncoprotein E7 from the “high-risk” Human Papillomavirus 16 (HPV16) strain is able, when expressed in human keratinocytes, to physically interact with the actin severing protein gelsolin (GSN). In a previous work it has been suggested that this protein-protein interaction can hinder GSN severing function, thus leading to actin network remodeling. In the present work we investigated the possible implications of this molecular interaction in cancer cell metastatic potential by analyzing two different human CC cell lines characterized by low or high expression levels of HPV16 DNA (SiHa and CaSki, respectively). In addition, a HPV-null CC cell line (C-33A), transfected in order to express the HPV16 E7 oncoprotein as well as two different deletion mutants, was also analyzed. We found that HPV16 E7 expression level was directly related with cervical cancer migration and invasion capabilities and that these HPV16 E7-related features were associated with Epithelial to Mesenchymal Transition (EMT) processes. These effects appeared as strictly attributable to the physical interaction of HPV16 E7 with GSN, since HPV16 E7 deletion mutants unable to bind to GSN were also unable to modify microfilament assembly dynamics and, therefore, cell movements and invasiveness. Altogether, these data profile the importance of the physical interaction between HPV16 E7 and GSN in the acquisition of the metastatic phenotype by CC cells, underscoring the role of HPV16 intracellular load as a risk factor in cancer.

## INTRODUCTION

The “high-risk” genotypes of Human Papillomaviruses (HPVs) are the most important etiological factors in squamous cervical carcinoma (CC). Along with this tumor, in which HPV infection is causatively correlated with virtually all cases, other cancers, as oropharyngeal squamous cell carcinoma (OSCC), anal carcinoma and non-melanoma skin cancer are presently considered, to some extent, as HPV-related [1–3].

Most of the HPV-induced tumors are characterized by the persistent expression of viral oncogenes and their protein products. These oncoproteins can reprogram fundamental cellular functions, thus generating a significant, partially explored, imbalance in cellular protein molecular networks and cell signaling pathways. All these changes appear finalized to a successful infection and include the survival of the infected cell, but, in a small percentage of cases, they can also favor the selection and rise of transformed cell clones [4–7]. The E7 oncoprotein, mainly from high-risk strains, is well known for its ability of hijacking normal cell physiology by weakening cell cycle control mechanisms and reducing cellular apoptotic response. Having no known enzymatic activity, E7 is able to reprogram fundamental cellular functions via specific protein-protein interactions it establishes with a large number of cellular factors [8–10].

In a previous paper, we described the physical interaction between the E7 oncoprotein from the high-risk HPV16 strain (HPV16 E7, henceforth E7) and the actin-binding protein gelsolin (GSN) [11], one of the most potent members of the actin-severing gelsolin/villin superfamily. GSN acts as a key regulator of actin filament assembly and disassembly, binds to the barbed ends of actin filaments and prevents monomer exchange (end-blocking or capping). Since actin can be cross-linked into a gel, GSN can turn this gel into a sol, hence the name “gelsolin”. Moreover, GSN has also been suggested to exert multifunctional roles inside the cell, functioning as transcription [12] or apoptosis regulator [13, 14].

In HaCaT immortalized human keratinocytes, a well-known model for E7 expression, the interaction between E7 and GSN results responsible for the alteration of the balance between polymeric (F-) actin and monomeric “globular” (G-) actin, causing an imbalance of the F-/G-actin ratio in favor of the former. The importance of this interaction stems from the key role played by actin microfilament network integrity and function. In fact, it has been suggested that “cell movements”, as migration and invasion ability and metastatic aggressiveness, could be under control of the microfilament network [15]. Moreover, in this context, the role of the E7 oncoprotein in promoting Epithelial-to-Mesenchymal Transition (EMT), a process that cancer cells employ to gain migratory and invasive properties and involves a dramatic reorganization of the actin cytoskeleton [16], has been suggested [17, 18].

Aim of the present work was to investigate the role of the physical interaction between E7 and GSN in an appropriate cancer cell context. To this end, two different human CC cell lines were considered herein: a low HPV16-expressing cell line (SiHa, 2 copies of HPV16 DNA per cell) and a high HPV16-expressing cell line (CaSki, 600 copies of HPV16 DNA per cell) [19]. The C-33A CC cell line, known as virtually devoid of any viral expression [20], was used as reference and for the unbiased expression of different E7 constructs. Our results indicated that the expression of E7 was able to modify migration and invasive capabilities of CC cells and that the mechanism underlying this behavior was apparently due to the molecular interaction between E7 and GSN. Importantly E7, although representing *per se* a pro-metastatic determinant, appeared to act in a dose-dependent manner, being its amount of expression directly correlated with CC cell “aggressiveness”.

## RESULTS

### E7 expression in CC cell lines

The present work was aimed at assessing whether the presence and the expression level of HPV16 could be relevant for carcinoma cells behavior and, in particular, the specific role of the E7 oncoprotein in the acquisition of a more malignant, pro-metastatic phenotype. First, we characterized three paradigmatic CC cells, the HPV-null C-33A [20] and the SiHa and CaSki cell lines (with low and high HPV16 DNA expression, respectively) [19], finding that these cell lines also expressed different levels of E7: null, low, or high, respectively, as measured by cytofluorimetric analysis (Supplementary Figure S1A, graph on the left), intensified video microscopy (IVM) analysis (Supplementary Figure S1A, micrographs on the right) and Western blot followed by densitometric quantification normalized against the expression of α-tubulin (Supplementary Figure S1B).

### HPV16 DNA expression correlates with actin cytoskeleton remodeling in CC cells

**Figure 1:**
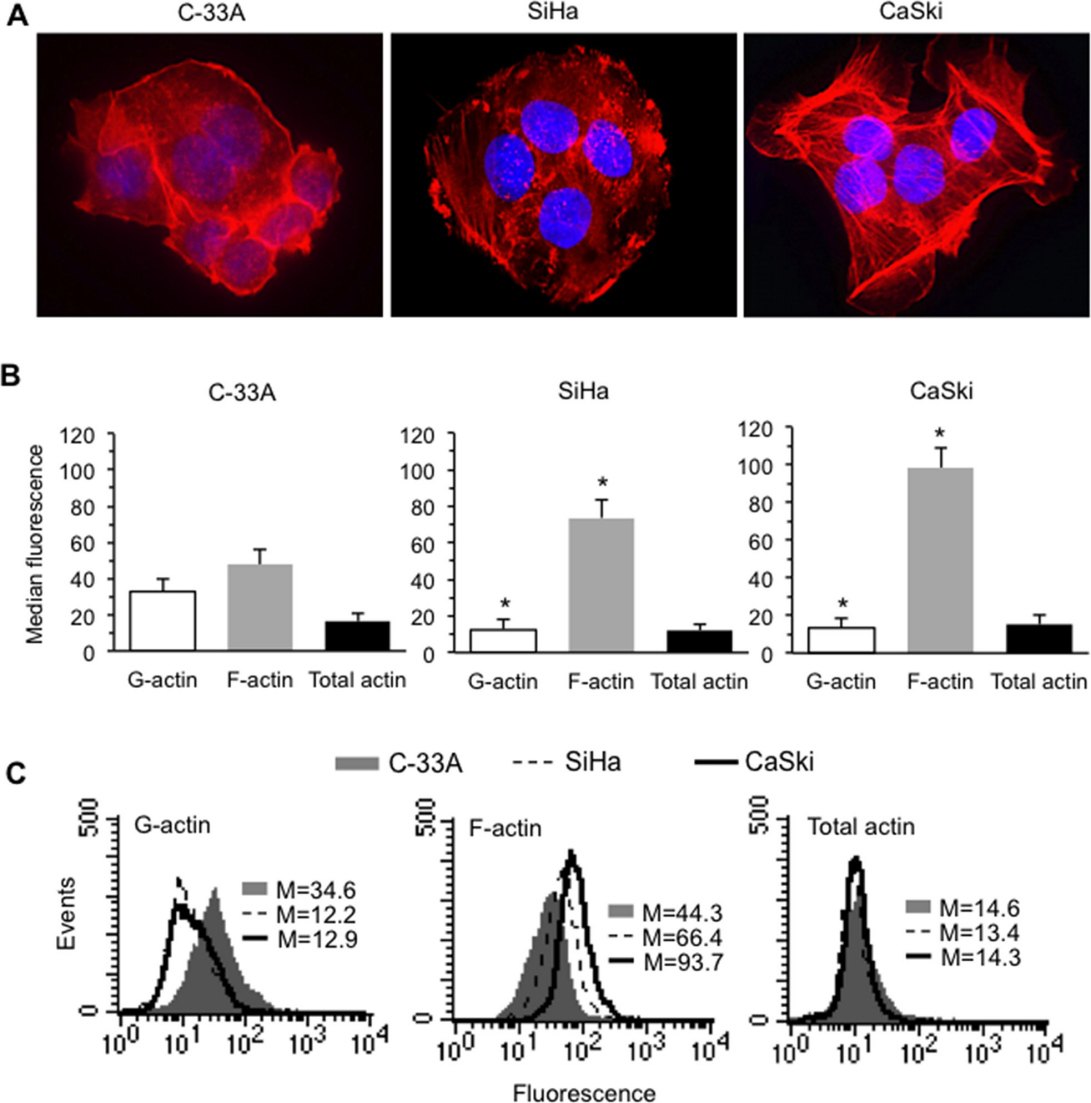
HPV16 DNA expression and actin cytoskeleton remodeling in CC cells (**A**) IVM analysis after TRITC-phalloidin/Hoechst double cell staining. Magnification, 700 ×. (**B**) Bar graphs showing the semi-quantitative flow cytometry analysis of intracellular amount of G-actin, F-actin and total (G + F) actin in C-33A (left panel), SiHa (central panel), and CaSki (right panel). Mean ± SD of the median fluorescence intensity obtained in four different experiments is reported. (**C**) Flow cytometry histograms obtained in a representative experiment are shown. Numbers represent the median fluorescence intensity. (*) indicates *P* < 0.01 *vs.* the corresponding bar of C-33A.

In light of our previous data, we evaluated the cellular amount of total actin (by a specific antibody) as well as its monomeric (G-actin, by DNAse I) and polymeric (F-actin, by phalloidin) forms, and the overall morphology of the above CC cell lines. We found different morphological features of microfilament network among the three cell lines (Figure 1A) and a different F-actin amount, which appeared strictly related to the different levels of HPV16 or E7 expression (Figure 1B and 1C). Accordingly, morphometric analyses clearly displayed a significant difference in terms of number of F-actin stress fibers, higher in CaSki cells, indicating a significant cytoplasmic remodeling in association with levels of HPV16 or E7 expression (Table 1).

**Table 1:** Morphometric analysis

Cell line	Stress fibers per cell	N/C ratio
C-33A	3 ± 1	0.6
SiHa	13 ± 3	0.28
CaSki	20 ± 4	0.26

Morphometric evaluation of two structural cell parameters - number of stress fibers and nucleus/cytoplasm ratio - in three different carcinoma cell lines that express different levels of HPV and E7 DNA: null (C-33A), low (SiHa) or high (CaSki). Of note, significant differences were appreciated between HPV16-null cells and cells with a different number of copies of HPV16 DNA, which show a higher content of polymeric actin stress fibers and also display a larger cytoplasm.

### HPV16 DNA expression correlates with Rho GTPases activation and increased cell invasion capability

Actin cytoskeleton is dynamically regulated by small GTPases of the Rho family [21]. In particular, Rho GTPases, through the action of their downstream effector proteins, drive actively cell migration and invasion [22].

**Figure 2:**
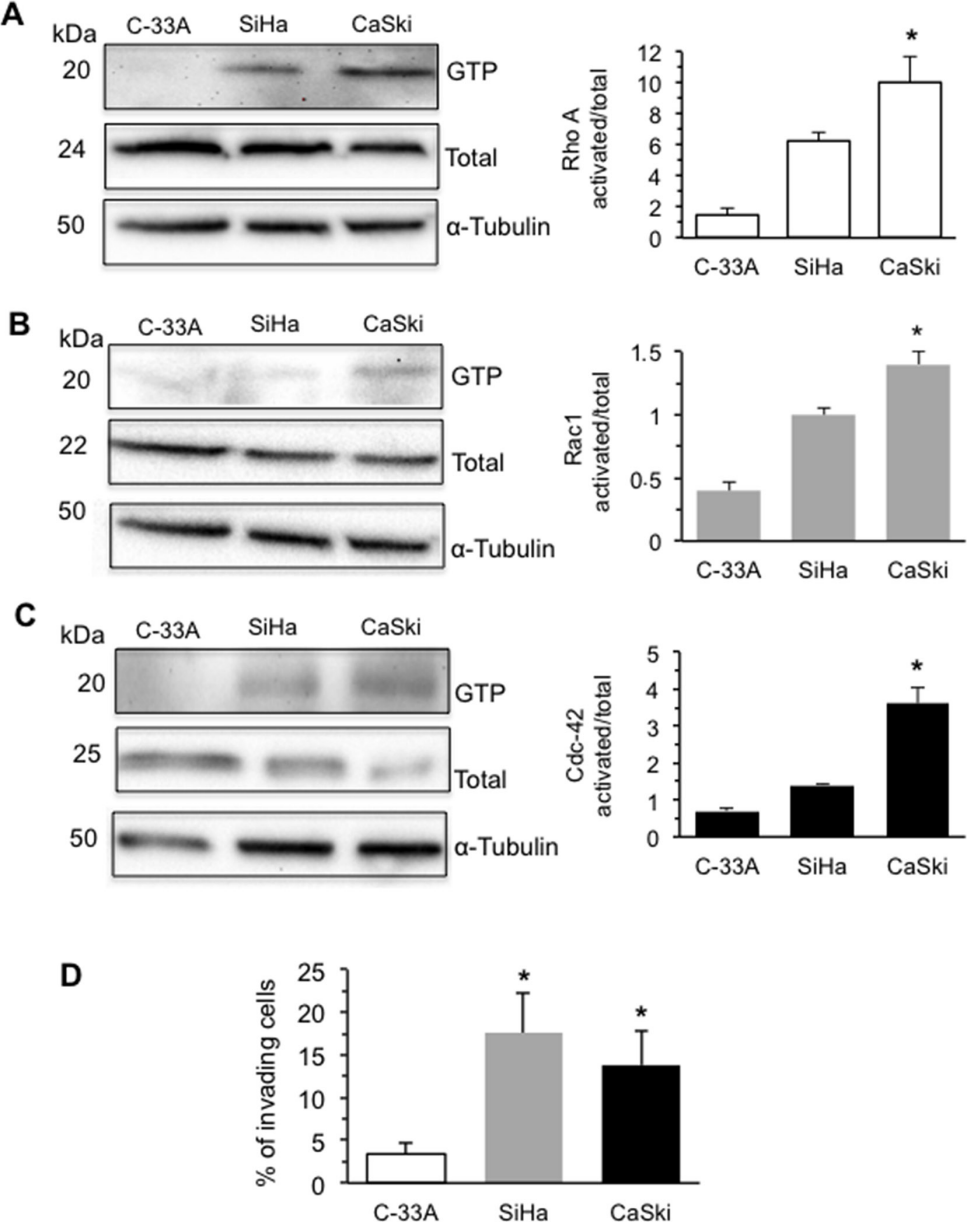
HPV16 DNA expression and activation of Rho GTPases and increases cell invasion Rho GTPase activation in human CC cells C-33A (E7-null cells), SiHa (2 copies of HPV16 DNA per cell), and CaSki (600 copies of HPV16 DNA per cell). Activation was measured by pull-down assays using the RBD domain of Rhotekin for (**A**) RhoA or the PBD domain of PAK for (**B**) Rac1 or (**C**) Cdc-42, followed by immunoblotting with the respective antibodies. Additionally, RhoA, Rac1, or Cdc-42 from total lysates was used as loading controls. In the right panels bar graphs show the active forms of RhoA, Rac1, and Cdc-42 GTPase (GTP-bound levels/total levels). The mean ± SD of the results obtained in three independent experiments is shown. (**D**) Invasion test on C-33A, SiHa and CaSki cell lines performed *in vitro* by using transwell culture inserts (8.0-μm pore size) coated with Matrigel. Data are reported as mean ± SD of the percentage of invading cells obtained in three independent experiments. (*) Indicates *P* < 0.01 *vs.* C-33A.

Therefore, we analyzed the activation of the best-characterized members of Rho family GTPases: RhoA, Rac1 and Cdc-42 in C-33A, SiHa and CaSki cell lines (Figure 2). We found that the GTP-bound active forms of RhoA (Figure 2A) and Rac1 (Figure 2B) were significantly higher in HPV16 DNA expressing SiHa and CaSki cells. By contrast, activated Cdc-42 was found significantly increased in CaSki cells only, those with the highest HPV16 DNA expression. In accordance with these data, either CaSki or SiHa cells showed a significantly higher ability to cross through Matrigel when compared with C-33A cells (*P* < 0.01 *vs.* C-33A) (Figure 2D).

### E7 co-localizes and interacts with GSN in CC cells

**Figure 3:**
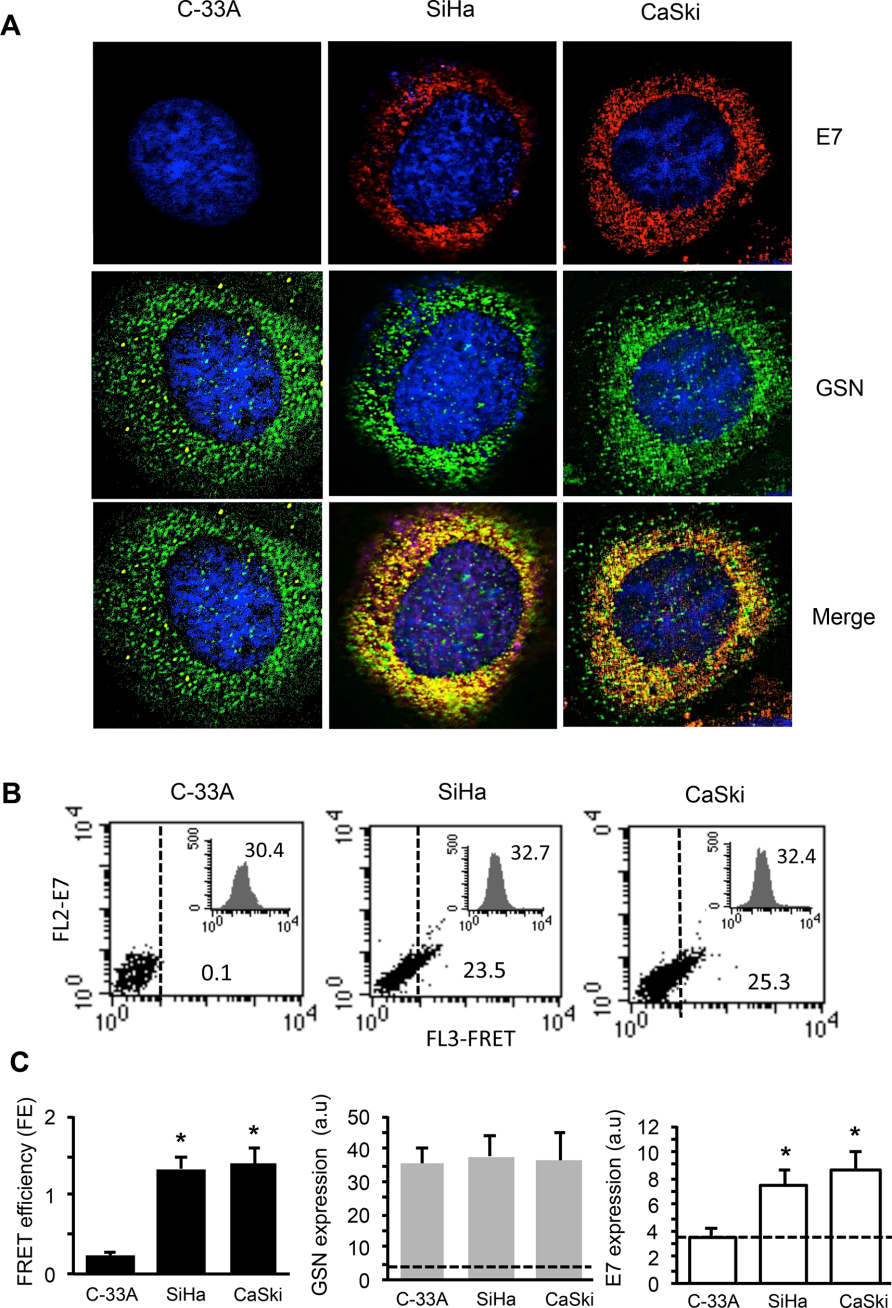
E7 interacts with GSN in carcinoma cells (**A**) IVM analysis after triple staining with anti-GSN/anti-E7/Hoechst in C-33A, SiHa and CaSki cells. Yellow fluorescence areas indicate the co-localization. Magnification 1,500×. (**B**) Quantitative evaluation of GSN/E7 association by FRET technique, as revealed by flow cytometry analysis. Numbers indicate the percentage of FL3-positive events obtained in one experiment representative of three. Inserts represent GSN intracellular amount in the corresponding sample and was quantitatively expressed by the median fluorescence intensity. (**C**) *Left panel*. Bar graph showing the evaluation of FE of GSN/E7 association. Numbers represent the FRET efficiency (calculated by using Riemann's algorithm) and are reported as the mean ± SD of data obtained in three independent experiments. *Central panel* and *right panel* show the mean ± SD of GSN and E7 intracellular amount, respectively, obtained from three independent experiments and quantitatively expressed by the median fluorescence intensity. Dashed lines designate the median fluorescence value of a negative control (baseline). (*) Indicates *P* < 0.01 *vs.* C-33A cells.

GSN is a cytoskeletal protein that participates in actin filament dynamics [23] also promoting cell motility. On this basis, and in the light of our previous results [11], we assessed, by means of IVM analysis and Fluorescence Resonance Energy Transfer (FRET), the occurrence of a protein-protein interaction between E7 and GSN. The results obtained by IVM (Figure 3A) clearly showed a co-localization (yellow staining in “merge” micrograph) of E7 with GSN in SiHa (second column) and CaSki cells (third column), which was undetectable in the E7 negative C-33A cells (first column). Accordingly, quantitative FRET analysis performed by flow cytometry, whose sensitivity allows performing a cell-by-cell analysis, revealed a significant interaction between GSN and E7 in SiHa and CaSki cell lines only (Figure 3B). This was further confirmed by FRET efficiency (FE) calculation performed by Riemann's algorithm [24] pooling together results obtained from three independent experiments (Figure 3C, left panel, black columns). Flow cytometry analysis also revealed that the expression level of GSN in C-33A, SiHa and CaSki was not significantly different (Figure 3C, central panel, grey columns). In the third panel of Figure 3C (white columns) the expression levels of E7 in the three cell lines are shown.

### Effects of transduced E7 in HPV-null C-33A CC cells

To study the effects of the expression of the sole E7 oncoprotein, HPV-null C-33A cells were transfected in order to express the wild type E7 oncoprotein (E7 wt) or one of its deletion mutants E7Δ62–66 and E7Δ71–75, both characterized for their impaired ability to physically interact with the GSN molecule [11].

**Figure 4:**
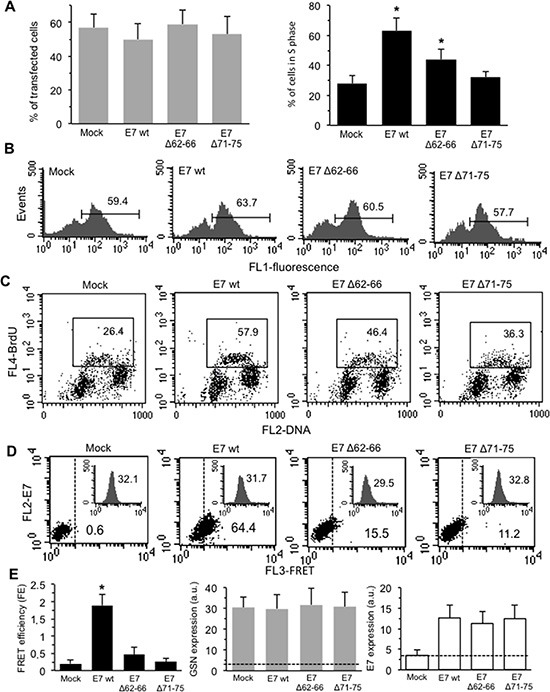
Effects of transduced E7 in HPV-16-null C-33A CC cells The HPV16-null C-33A cells were transfected with E7 oncoprotein (E7 wt) or one of its deletion mutants E7Δ62-66 and E7Δ71-75. (**A**) *Left panel.* Bar graph showing the mean ± SD of the percentages of transfected cells (FL1 positive cells) obtained by flow cytometry in three different experiments. *Right panel.* Bar graph showing the mean ± SD of the percentages of transfected cells in the S phase of the cell cycle (FL1/FL4 double positive cells) obtained by flow cytometry analysis in three different experiments. (**B**) Flow cytometry histograms of transfected cells obtained in a representative experiment. Numbers represent the percentages of FL1 positive cells. (**C**) Biparametric flow cytometry analysis of the cell cycle restricted to transfected (FL1 positive) cells only. The y axis depicts the degree of BrdU fluorescence: cells actively synthesizing DNA incorporate BrdU (S phase of the cell cycle); the x axis portrays PI fluorescence. Numbers into the boxes indicate the percentage of the transfected cells in S phase of the cell cycle. Representative dot plots are shown. (**D**) Quantitative evaluation of GSN/E7 association by FRET technique, as revealed by flow cytometry analysis in FL1 positive cells (transfected). Numbers indicate the percentage of FL3-positive events obtained in one experiment representative of three. Inserts represent GSN intracellular amount in the corresponding sample and was quantitatively expressed by the median fluorescence intensity. (**E**) *Left panel*. Bar graph showing the FE, calculated according to the Riemann's algorithm, of GSN/E7 association. Data are reported as mean ± SD from three independent experiments. *Central panel* and *right panel* show the mean ± SD of GSN and E7 intracellular amount, respectively, obtained from three independent experiments and quantitatively expressed by the median fluorescence intensity. Dashed lines designate the median fluorescence value of a negative control (baseline). (*) Indicates *P* < 0.01 *vs.* mock-transfected cells.

In this set of experiments, our analyses were restricted to the cells effectively transfected (Supplementary Figure S2). The efficiency of the transfection procedure ranged from 40.7 to 65.1% for all the E7 constructs employed (Figure 4A, left panel, grey columns, average values).

#### Cell cycle

Results obtained by biparametric flow cytometry analysis are shown in Figure 4A (bar graph, right panel, black columns, mean values from three independent experiments). In Figure 4B, flow cytometry plots indicate that the percentage of transfected cells in a representative experiment was similar in all samples analyzed. In the same sets of experiments, according with literature data [8, 9, 25], we observed, after transfection with E7 wt, a significant increase in the percentage of BrdU-positive, S phase cells (in Figure 4C, second panel, a representative experiment is reported). These results are in accord with morphometric evaluations shown in Table 1. Either E7Δ62–66 or E7Δ71–75 mutants retained some effects on the cell cycle (greater in E7Δ62–66), although not comparable with those of E7 wt (Figure 4A, right panel and Figure 4C).

#### Interaction with GSN

FRET technique highlighted a physical interaction with GSN only when E7 wt was expressed, whereas the two E7Δ62–66 and E7Δ71–75 mutants were almost completely unable to interact with GSN (Figure 4D and 4E, left panel, black column). Flow cytometry analysis also revealed that the expression level of GSN was comparable in all samples analyzed (inserts in Figure 4D and Figure 4E, central panel, grey columns). In the right panel of Figure 4E (white columns) the expression levels of E7 in the C-33A cell line, either wild-type or mutant, are shown.

#### Actin cytoskeleton

**Figure 5:**
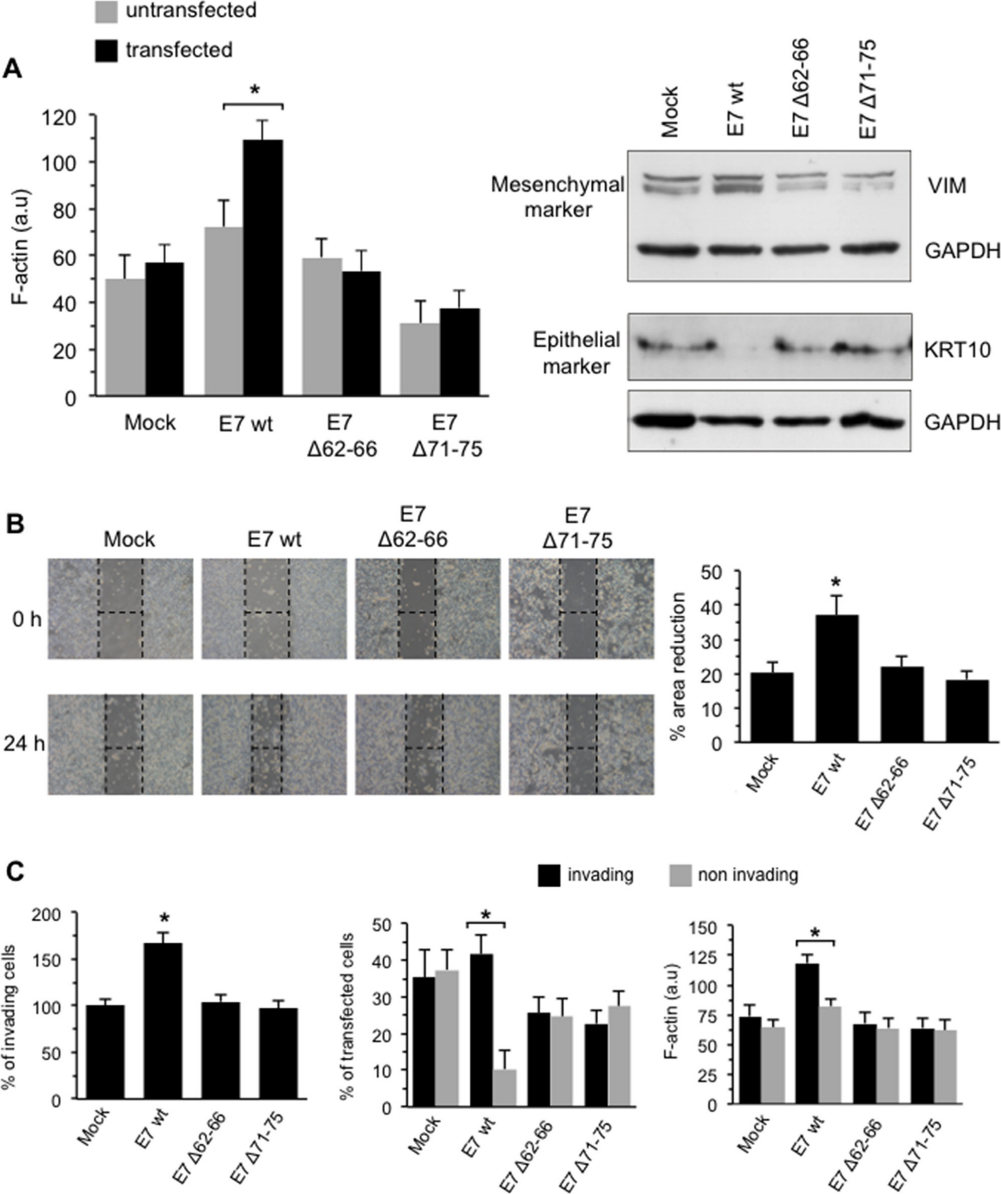
Effects of transduced E7 in HPV-16-null C-33A CC cells (**A**) *Left panel*. Flow cytometry evaluation of F-actin intracellular content (expressed by the median fluorescence intensity in FL4 channel) in transfected (FL1/FL4 double positive cells, black columns) or non-transfected (FL4 single positive cells, grey columns) cells. Results are reported as mean ± SD among three independent experiments. *Right panel*. Western blot analysis of vimentin (VIM) and keratin 10 (KRT10) in C33-A transfected cells. Loading control was evaluated using anti-α-tubulin MAb. (**B**) *Right panel*. Migration test performed by scratch assay on C-33A transfected with mock construct, with E7 (E7 wt) or one of its deletion mutants E7Δ62-66 and E7Δ71-75. Representative phase contrast microscopy images of three independent experiments are shown. *Left panel.* Quantification of the area reduction at 24 h represented as the percentage of wounded area at time 0. Results represent the mean ± SD of three independent experiments. (*) Indicates *p* < 0.01 *vs.* E7wt-transfected cells. (**C**) *Left panel*. Invasion test on C-33A cells transfected with E7 oncoprotein (E7 wt) or one of its deletion mutants E7Δ62-66 and E7Δ71-75 performed *in vitro* by using transwell culture inserts (8.0-μm pore size) coated with Matrigel. Data are reported as mean ± SD of the percentage of invading cells obtained in three independent experiments. *Central panel*. Flow cytometry differential analysis of invading (bottom Matrigel-coated filter) or non-invading (upper Matrigel-coated filter) cells to quantify the percentage of transfected (FL1 positive) cells. *Right panel.* Flow cytometry evaluation of F-actin intracellular content (expressed by the median fluorescence intensity) in invading or non-invading cells. Results are reported as mean ± SD obtained from three independent experiments. Analyses reported in (C) were restricted to transfected (FL1 positive) cells only. (*) Indicates: *p* < 0.01 *vs.* E7 wt-transfected cells in (B) and (C), left panel; *P* < 0.01 *vs.* the indicated sample in (A) and (C), central an right panels.

A differential analysis performed in non-transfected (fluorescence-negative) and transfected (fluorescence-positive) cells revealed that, when compared with mock-transfectants, E7 wt-expressing C-33A cells exhibited an overall increased amount of F-actin, as demonstrated by the significant increase in phalloidin fluorescence emission observed 72 h after transfection. At variance, C-33A cells transfected with the E7Δ71–75 mutant displayed a considerably lower F-actin amount, while the E7Δ62–66 mutant gave intermediate results (Figure 5A, bar graph in the left panel). The results obtained by expressing both E7 mutants unable to bind to GSN (see above, Figure 5A) apparently confirmed that the interaction of E7 with GSN represents a critical trigger for the remodeling of the actin cytoskeleton.

### EMT

In the same C-33A cell model, mock-, E7 wt-, E7Δ62-66- or E7Δ71–75-transfected cells, the expression of EMT markers was analyzed by Western blot. As shown in Figure 5A, E7 wt-transfected C-33A cells displayed increased levels of vimentin (VIM), a mesenchymal marker, and very low levels of cytokeratin-10 (KRT10), an epithelial marker, when compared with mock, E7Δ62–66 or E7Δ71–75 transfectants. Glyceraldehyde-3-phosphate dehydrogenase (GAPDH) determination was used as a loading control. These results confirmed the ability of E7 to trigger a complex cytoskeleton remodeling involving either microfilament network, essential for a number of cell activities including movements, or intermediate filament vimentin network, which is essential for the acquisition of a mesenchymal phenotype [17, 18], loss of desmosomal contacts, increase in focal adhesion dynamics and cell motility. Conversely, the two E7 mutants failed to drive these processes.

#### Cell motility

C-33A cells, transfected with a mock construct or with constructs expressing E7, either wt or mutants, were then subjected to an *in vitro* scratch assay [26]. Data were collected 24 h following the induction of the lesion. We found that E7 wt was able to significantly accelerate the repair of the induced lesion, whereas the effects of the two mutants unable to interact with GSN were negligible (representative phase contrast micrographs are shown in Figure 5B). This was confirmed by statistical analyses performed pooling together the results of three independent experiments (Figure 5B, bar graph in the right panel).

#### Cell invasion

The same C-33A model used to assess cell motility was also employed to assay the capability of these cells to pass through a Matrigel membrane (Figure 5C). In this setup, expression of E7 wt increased significantly the number of invading cells, whereas the two mutants gave results overlapping those observed using the mock construct (Figure 5C, left panel). A further very important result was obtained assessing whether the cells that invaded the barrier were actually those E7 wt-transfected. In fact, a high percentage of cells migrated (below the Matrigel-covered filter) corresponded to those in which transfection occurred (fluorescence-positive) (Figure 5C, central panel). Interestingly, unlike mutant-transfected cells, E7 wt-transfected cells below the Matrigel-covered filter also displayed a significantly higher F-actin content (Figure 5C, right panel). Furthermore, when compared with the two mutants, the specificity of the effect of E7 wt on the induction of cell migration (Figure 5B) and invasion (Figure 5C) strengthens once again the hypothesis that the physical interaction between E7 and GSN could induce a cascade of events in cytoskeletal network, increasing the overall cell motility and migration of C-33A carcinoma cells.

### E7 down-regulation by siRNA hinders the acquisition of the metastatic phenotype

At this point, we down-regulated the expression of endogenous E7 by means of a specific siRNA [27]. These experiments were performed in the “counterpart” of C-33A cells, namely in those cell lines that display low (SiHa) or high levels (CaSki) of HPV16 DNA. This gave us the opportunity to evaluate functional changes specifically related to the sole E7 oncoprotein down-regulation. In these experiments, the efficiency of FITC-labeled siRNA transfection procedure ranged between 59% and 83%, as determined by flow cytometry. In these experimental conditions, we obtained a down-regulation of endogenous E7 protein expression level between 60 and 75%, as evaluated by flow cytometry (Supplementary Figure S2).

### EMT

**Figure 6:**
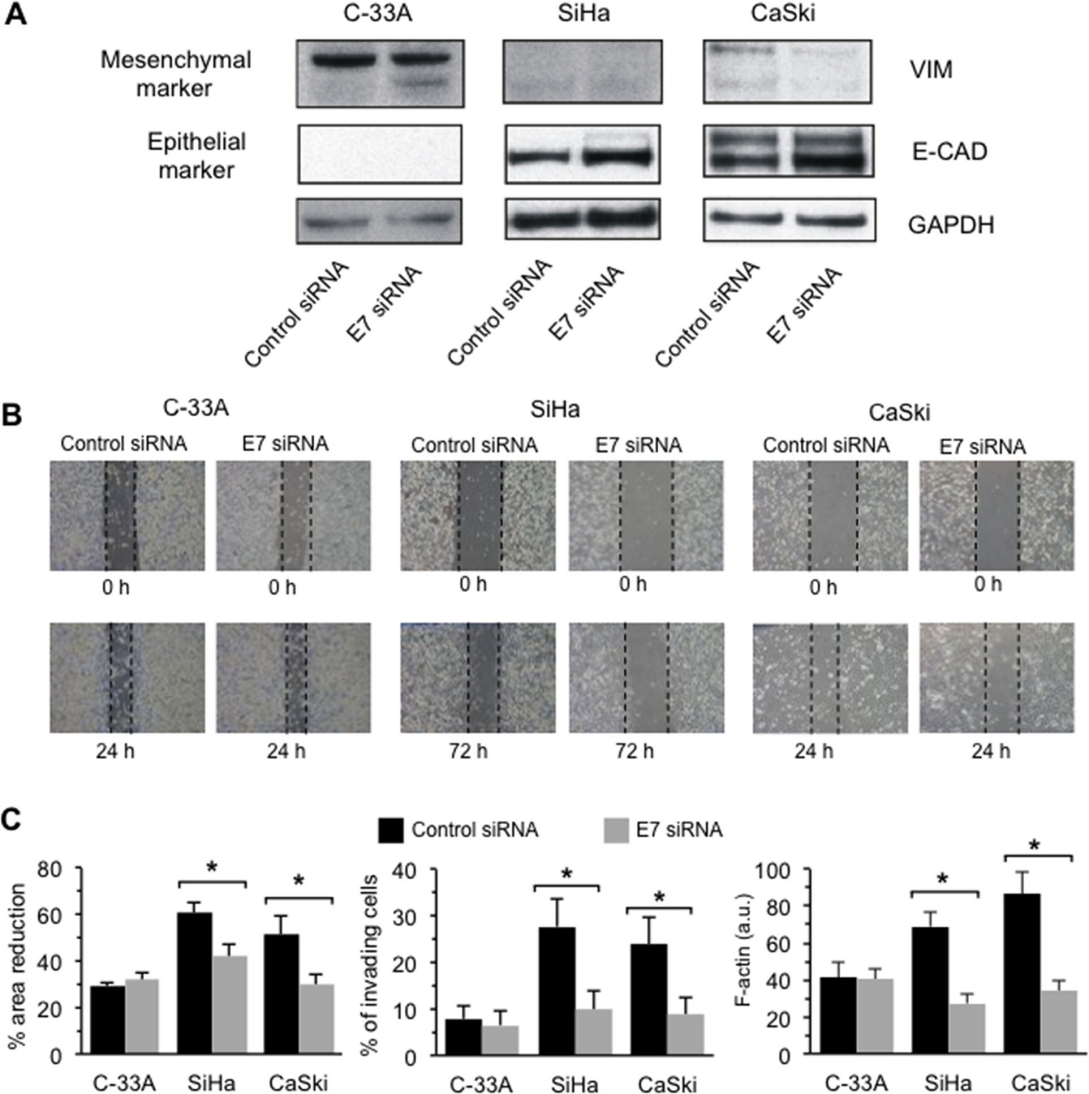
E7 down-regulation by siRNA hinders the acquisition of the metastatic phenotype (**A**) *Left panel.* Western blot analysis of vimentin (VIM), E-cadherin (E-CAD) on C-33A, SiHa and Caski cells transfected with control siRNA and E7 siRNA. Loading control was evaluated using an anti-GAPDH MAb. (**B**) Migration test performed by scratch assay on C-33A, SiHa and Caski cells transfected with control siRNA or with E7siRNA. Images were captured by phase-contrast microscopy using a 4.6 × objective at 0, 24/72 h post wounding. A representative experiment among three is shown. (**C**) *Left panel* Quantification of the area reduction represented as the percentage of wounded area at time 0. (*) Indicates *p* < 0.01 between cells transfected with control siRNA and E7 siRNA in the indicated sample. *Central panel.* Invasion test on C-33A, SiHa and CaSki cell lines performed *in vitro* by using transwell culture inserts (8.0-μm pore size) coated with Matrigel after E7 silencing by siRNA. Data are reported as mean ± SD of the percentage of invading cells obtained in three independent experiments. *Right panel*. Flow cytometry evaluation of F-actin intracellular content (expressed by the median fluorescence intensity) in control siRNA-transfected or in E7 siRNA-transfected cells. Results are reported as mean ± SD obtained from three independent experiments. (*) Indicates *P* < 0.01 between cells transfected with control siRNA and E7 siRNA in the indicated samples.

C-33A, SiHa and CaSki cell lines were transfected with control or E7-specific siRNA and were analyzed by Western blot for the expression of EMT markers (Figure 6A). E7 silencing in CaSki cells induced a down-regulation of the mesenchymal markers vimentin (VIM). In SiHa cells, in which VIM expression was undetectable, a substantial down-regulation of fibronectin (FN), a further well-known EMT marker, was detected (Supplementary Figure S4). C-33A cells, due to their HPV-null status (i.e. in the absence of the siRNA target), showed a negligible interference with VIM expression. Accordingly, the expression of the epithelial marker E-cadherin (E-CAD) was higher in both E7-silenced SiHa and CaSki cell lines. GAPDH determination was used as a loading control (Figure 6A). All in all, these data suggested that silencing of E7 is able to partially revert EMT, thus enforcing the hypothesis that this oncoprotein could be a key inducer of EMT [17, 18].

#### Cell motility

We then performed a scratch assay [26] in HPV16 DNA expressing SiHa and CaSki CC cell lines when E7 expression was silenced by specific siRNA [27]. HPV-null C-33A cells were also assayed in order to provide an internal control. Data on cell migration were collected after 24 h for C-33A and CaSki cell lines, and after 72 h for the SiHa cell line that exhibited a constitutively lower motility. In Figure 6B, representative phase contrast micrographs are shown. Statistical analyses performed pooling together results obtained in three independent experiments showed that the siRNA-specific E7 down-regulation significantly decreased SiHa and CaSki cell lines motility. No significant effects were detected on the C-33A HPV-null cell line (Figure 6C, left panel).

#### Cell invasion

We also found that, when E7 expression was down-regulated by specific siRNA, either SiHa or CaSki cells showed a significantly reduced ability to cross the Matrigel barrier when subjected to an *in vitro* invasion assay test (Figure 6C, middle panel). According with this, and with above reported data, we found a parallel significant reduction of F-actin in SiHa and CaSki cells transfected with E7 siRNA in comparison to cells transfected with control siRNA (Figure 6C, right panel).

## DISCUSSION

Besides CC, in which high-risk HPVs are implicated in virtually all cases, these small DNA viruses appear now deeply involved in the genesis of many other human cancers. HPV16 genotype, in particular, is found in roughly half of cervical cancers and its role in OSCC is also well established [1–3]. Thus, in view of the growing pathogenetic relevance of this viral strain, an extensive analysis of the implication of a key protein expressed early in the HPV life cycle, the E7 oncoprotein, in the acquisition of a pro-metastatic phenotype has been investigated. The data here reported strongly suggest that, besides its transforming properties, E7, *via* its molecular interaction with GSN, could ignite *per se* a pro-metastatic phenotypic remodeling of epithelial cells. We found that the expression of the E7 oncoprotein can in fact generate a significant remodeling of the actin filament network and trigger EMT, the process thru which epithelial cells modify their polarity and adhesion properties acquiring migratory and invasive capabilities.

It has already been suggested that E7 could enhance cytoplasmic retention of p27, a cyclin-dependent kinase inhibitor, thus removing constriction to cell division and enhancing cell migration in E7-expressing keratinocytes [28]. Moreover, in a comparable keratinocyte cell model, we demonstrated that E7 can bind GSN and hinder its severing function, thus increasing the intracellular level of polymeric actin [11]. Accordingly, E7 has also been implicated in microfilament cytoskeleton integrity and function by acting on Rho GTPases, a family of small GTPases (e.g. Rho, Rac1 and Cdc42) involved in a plethora of cell features, including adhesion or, conversely, spreading, and controlling a wide variety of signal transduction pathways [29]. Here, we established that the physical interaction between E7 and GSN occurs also in CC cells, leading to a more aggressive phenotype, displaying increased migration and invasion ability, two features with key implications in cancer progression and metastasis. Furthermore, these features appeared, in our experimental models, somehow related to the number of copies of the E7 oncoprotein, where to higher E7 expression corresponded higher metastatic potential, either in terms of cytoskeletal remodeling needed for migration and invasion ability or in terms of EMT markers expression. Conversely, when E7 was down-regulated via a specific siRNA, this cell remodeling was hindered. On the other hand, the C-33A HPV-null cell line, when forced to express E7, clearly acquired migrating and invading as well as EMT features. Altogether, these analyses clearly suggest that the molecular association between GSN and E7, here re-assessed by different approaches, including FRET, could ignite (or contribute to) the complex framework of events associated with the acquisition of the metastatic phenotype. Accordingly, we found that specific E7 deletion mutants unable to bind GSN were also unable to trigger the series of cell changes described above. These mutants, according to the 3D molecular interaction model previously hypothesized [11], are defective in the region responsible for the docking with GSN. Strikingly, high E7 expression, as in CaSki cell line, in comparison with its low expression, as in SiHa cell line, seems to represent an effective stimulus for the acquisition of a more malignant and metastasis-prone phenotype.

Although still controversial, some literature data suggest that E7 could act as a trigger of the complex framework of events leading to actin cytoskeleton reorganization, contributing to increase motility and metastatic spreading of cancer cells [29]. In line with this view, our results carve out a central role for E7 in determining cancer cell features, strictly related with malignancy grade and metastatic potential. In this context, our work points out the possibility that GSN could be a key factor in this cell behavior and that its physical association with E7 could increase cell movements, migration and invasiveness in HPV-related cancers. The fact that the transfection of E7 alone in HPV-null CC cells was able to elicit migration, invasion and possibly trigger a pro-metastatic behavior, similar to that of HPV16 DNA expressing cells, seems to reinforce this hypothesis and appears suggestive for a central role of E7 in cytoskeleton remodeling. In few words, although to be investigated in near future in animal models, our results strongly suggest that hindering of this interaction could significantly contribute to lower aggressiveness and metastatic potential in HPV16-related human cancers.

## MATERIALS AND METHODS

### Cell lines

CaSki (HPV-16+), SiHa (HPV-16+) and C-33A (HPV-) human CC cell lines were obtained from the American Type Culture Collection (ATCC, Rockville, MD). SiHa and C-33A cells were cultured in DMEM (Invitrogen Corporation, Carlsbad, CA) supplemented with 10% fetal bovine serum (Euroclone, Milan, Italy), while CaSki cell line was grown in RPMI 1640 medium (Invitrogen) containing 10% fetal bovine serum. All cell lines were incubated at 37°C in a humidified atmosphere of 5% (v/v) CO_2_ in air.

### Plasmids, siRNAs and transfections

Full-length wild-type and deletion mutants of E7 were generated as previously described [11].

The siRNAs used in this study (Eurofins Genomics, Ebersberg, Germany, all at 50 nM final concentration) were the following, according to Yamato et al. [27]:

- siRNAE7HPV16 sense 5′-CCGGACAGAGCCC AUUACAAU-3′
- siRNAE7HPV16 antisense 5′-AUUGUAAUGGGC UCUGUCCGG-3′
- ON-TARGETplus siCONTROL non-targeting control pool (D-001810-10 Dharmacon Inc., Lafayette, CO).

Plasmid transfections were performed using Lipofectamine 2000 (Invitrogen) according to manufacturer's instructions. siRNA transfections were done as previously described [30].

### Western blot analysis

SDS-PAGE and Western blot analysis with ECL detection were performed as described [31]. The following antibodies and dilutions were used: anti-E-cadherin MAb (BD Biosciences, Franklin Lakes, NJ, 1:2000); anti-vimentin PAb (Cell Signaling, Beverly, MA, 1:1000); anti-fibronectin MAb (EP5, Santa Cruz Biotechnology, Inc. Santa Cruz, CA, 1:500); anti-cytokeratin 10 MAb (Invitrogen, 1:5000); anti-E7 antibody MAb (Cervimax-Valdospan GmbH, Vienna, Austria, 1:500); anti-GAPDH MAb (Santa Cruz, 1:1000); anti-α-tubulin MAb (Calbiochem, Merck KGaA, Darmstadt, Germany, 1:500);. Quantification of protein expression was performed by ChemiDoc MP system (Bio-Rad, Hercules, California, USA).

### Determination of Rho GTPase protein activity

Activation of RhoA, Rac1 and Cdc-42 was determined with the Rho/Rac/Cdc-42 Activation Assay Combo Kit (Cell Biolabs, San Diego, CA, USA). The activation of Rho GTPases was assessed, after cell lysis, by Western blotting according to the manufacturer's protocol.

### Static cytometry analyses

#### Immunofluorescence microscopy

For triple fluorescence analysis, cells were fixed with 4% paraformaldehyde and then permeabilized by 0.5% (v/v) Triton X-100 as reported [32]. The following primary and secondary antibodies were used: mouse MAb anti-HPV16 E7 antibody (Cervimax), rabbit PAb anti-gelsolin (Novus Biologicals), AlexaFluor 488-conjugated anti-rabbit (Invitrogen), AlexaFluor 488-conjugated anti-mouse IgG (Invitrogen Corporation), and AlexaFluor 594-conjugated anti-mouse IgG (Invitrogen). For F-actin detection, cells were stained with TRITC-phalloidin (Sigma, St Louis MO, USA) for 30 min at room temperature. After washing, all samples were counterstained with Hoechst 33258 (Sigma, 1 mg/ml in PBS) and then mounted in glycerol/PBS (ratio 1:1, pH 7.4). The images were acquired by intensified video microscopy (IVM) with an Olympus fluorescence microscope (Olympus Corporation of the Americas, Center Valley, PA.), equipped with a Zeiss charge-coupled device (CCD) camera (Carl Zeiss, Oberkochen, Germany).

#### Morphometric analyses

Morphometric evaluations of two structural cell parameters - the number of stress fibers and the nucleus/cytoplasm ratio - in different carcinoma cell lines were carried out by evaluating at least 200 cells at the same magnification (1,300×) at the fluorescence microscope by using the IAS2000 software (Delta Sistemi, Milan, Italy).

### Flow cytometry analyses

#### Cytoskeleton analysis

Microfilament system dynamic was analyzed by quantification of filamentous actin (F-actin), globular actin (G-actin), and total (F + G) actin [33]. Cells fixed and permeabilized as above, were incubated at 37°C for 45 min with: FITC-DNase I (Sigma) for G-actin detection and Cy5-Phalloidin (Molecular Probes, Eugene, OR, USA) for F-actin detection. For total actin detection, cells were incubated with monoclonal antibody to actin (Santa Cruz) followed by an AlexaFluor 488-conjugated anti-mouse IgG (Invitrogen).

The median values of fluorescence intensity were used to provide semi-quantitative assessment of F-actin, G-actin and total actin.

#### Fluorescence resonance energy transfer (FRET)

We applied quantitative FRET analysis by flow cytometry, in order to study cell-by-cell the molecular association of E7 oncoprotein with GSN in different experimental conditions in entire cells. As previously reported [32] cells were fixed and permeabilized and then labeled with antibodies tagged with donor (Phycoerythrin, PE) or acceptor (Cy5) dyes. The following antibodies were used: anti-E7 mouse MAb antibody (Cervimax), anti-gelsolin rabbit PAb antibody (Novus Biologicals), PE-labeled anti-mouse (Sigma), and Cy5-labeled anti-rabbit (Termo Fisher Scientific, Waltham, MA USA). In particular, E7 protein was detected in the FL2 channel (PE), GSN in FL4 channel (Cy5), and FRET in FL3 channel.

FRET efficiency (FE) was calculated according to Riemann et al. [24] by using the following algorithm:

FE = [FL3DA – FL2DA/a – FL4DA/b]/FL3DA in which A is the acceptor and D the donor and where a = FL2D/FL3D and b = FL4A/FL3A.

#### Cell cycle analysis

To estimate cell proliferation, control and transfected cells were seeded in triplicate at 10^5^ cells per 35 mm dish. Briefly, 72 h after seeding cells pulse-labeled for 45 min with 30 μM of 5-bromo-2-deoxy-uridine (BrdU, Sigma). After this time cells were processed as previously reported and then analyzed for cell cycle by a biparametric flow cytometry analysis [34].

### Wound healing assay

Cell migration was examined by scratch assay according to Liang et al. [26]. Approximately 2.5 × 10^5^ cells were seeded in 35-mm dishes and transfected 24 h later. When transfected cells reached confluence, dishes were scratched with a sterile 200-μl pipette tip. After scratching, cells were washed three times with PBS and incubated at 37°C. Migration of cells towards wound closure of the same region at different times (0, 24, 48 or 72 h) was monitored, and images were acquired using a digital camera system coupled with an inverted microscope (Olympus IX-71). Repopulation by migrating cells of the wound region was then analyzed and quantified using the ImageJ v1.48 software (http://imagej.nih.gov/ij/).

### Invasion assay

Tumor cell invasion was determined *in vitro* by using transwell culture inserts (8.0-μm pore size) coated with Matrigel (BD) as previously reported [35]. Each assay was carried out at least three times in triplicate for each experimental condition. To determine the percentage of transfected cells or the F-actin content in cells invading and non-invading, cells on the filter top or migrating to the bottom surface of the filter were harvested and analyzed separately by a cytometer.

### Data analysis and statistics

For flow cytometry studies, all samples were analyzed by a dual-laser FACScalibur cytometer equipped with a 488 argon laser and with a 635 red diode laser. At least 20,000 events/sample were acquired. Data of the single flow cytometry experiments were recorded and statistically analyzed by a Macintosh computer using CellQuestPro Software.

Statistical analysis of collected data was performed using the two-tailed Student's *t* test or ANOVA two-way test, with GraphPad Prism v5.01 (GraphPad Software, San Diego CA). Values of *P* < 0.01 (*) were considered as statistically significant. All data reported in this paper were verified in at least three different experiments and reported as mean ± Standard Deviation (SD).

## SUPPLEMENTARY MATERIALS


