# Supplementary material for: Interaction between the human papillomavirus 16 E7 oncoprotein and gelsolin ignites cancer cell motility and invasiveness
Source: Oncotarget. 2016 Apr 8;7(32):50972–85. doi: 10.18632/oncotarget.8646 (PMC5239452; doi:10.18632/oncotarget.8646)
Supplement: Supplementary file 1 [file oncotarget-07-50972-s001.pdf]

Interaction between the human papillomavirus 16 E7 oncoprotein and gelsolin ignites cancer cell motility and invasiveness

Supplementary Materials

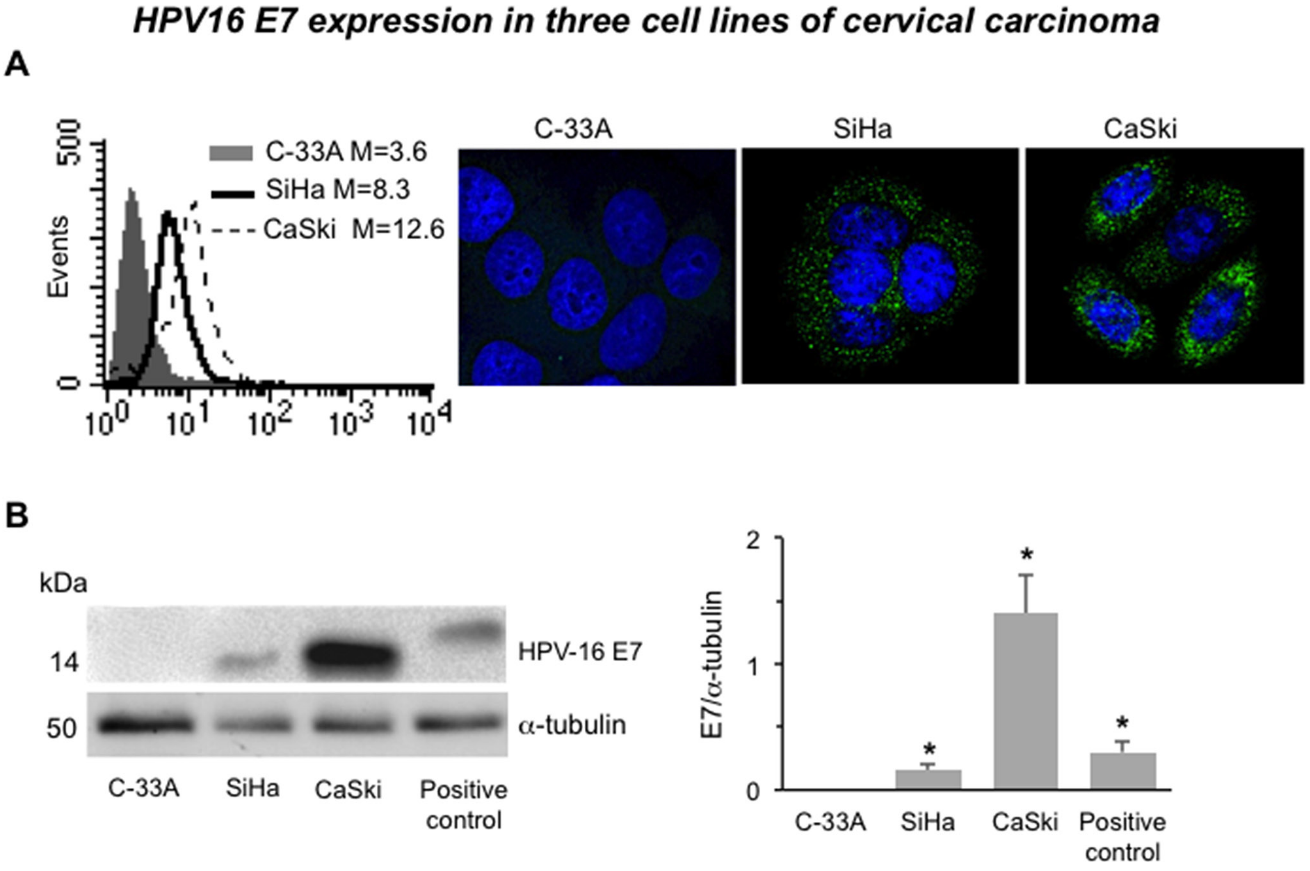

Supplementary Figure S1: E7 expression in CC cell lines. (**A**) *Left panel*. Cytofluorimetric analysis after single cell staining with anti-E7 MAb. Numbers represent the median fluorescence intensity obtained in a representative experiment. *Micrographs*. IVM analysis after anti-E7 MAb/Hoechst double cell staining. Magnification, 1,300×. (**B**) Western blot analysis using an anti-E7 MAb. E7 positive control cells displayed a protein with a higher apparent molecular mass due to the addition of a T7 tag sequence. Loading control was evaluated using anti-α-tubulin MAb. Density of each band in the same gel was analyzed and the densitometric E7/α-tubulin ratios are shown in the bar graph in the right panel. (*) indicates $P < 0.01$ *vs*. C-33A.

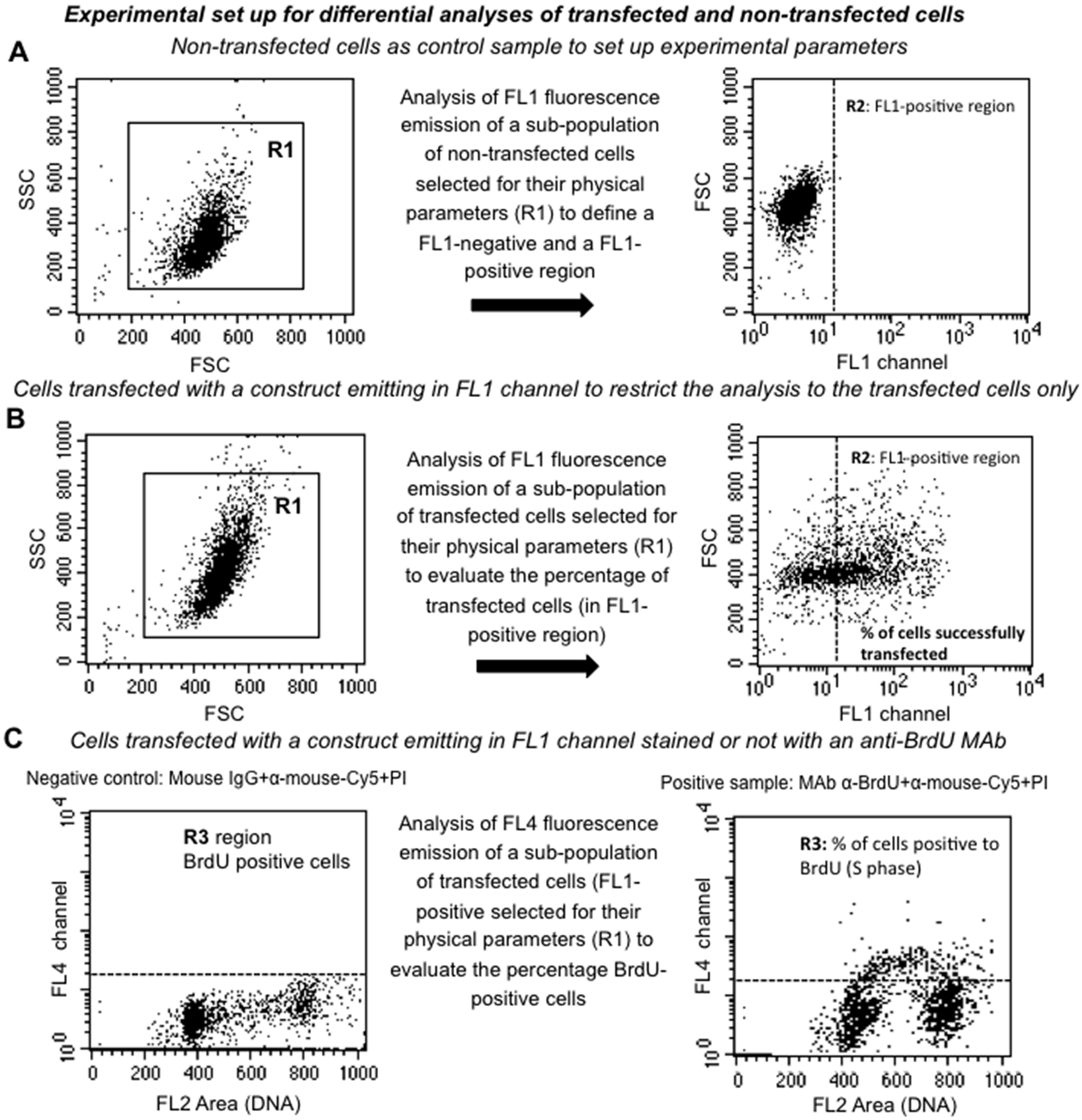

Supplementary Figure S2: Experimental set up for differential analyses of transfected and non-transfected cells. Exemplification of a flow cytometry analysis to differentiate transfected and non-transfected cells. (**A**) *Left panel*. Non-transfected cells were selected on the basis of their physic parameters (R1 region). *Right panel*. Analysis of FL1 fluorescence emission in R1 cells subpopulation to define FL1-negative and FL1-positive (R2) regions. (**B**) *Left panel*. Selection of a population of transfected cells on the bases of its physic parameters (R1). *Right panel*. FL1 fluorescence emission analysis of cells included in R1 region to quantify the percentage of actually transfected cells on the basis of FL1 negative and positive regions defined in non-transfected cells sample. (**C**) Seventy-two hours after transfection, cells were incubated with BrdU and then fixed and permeabilized as reported in Material and Methods. After this step, each experimental sample was divided into two: a negative control (IgG1 + anti-mouse Cy5-conjugated + PI) and a positive sample (MAb anti-BrdU + anti-mouse Cy5-conjugated + PI). *Left panel*. Multiparametric analyses of negative control (FL1, FL2 and FL4 fluorescence channels) to distinguish a FL4-negative and a FL4-positive region (R3). *Right panel*. Multiparametric analyses of a positive sample (FL1, FL2 and FL4 fluorescence channels) to evaluate the percentage of cells in FL4- positive region (R3, cells positive to anti-BrdU). Analysis was restricted to FL1 positive (transfected) cells only.

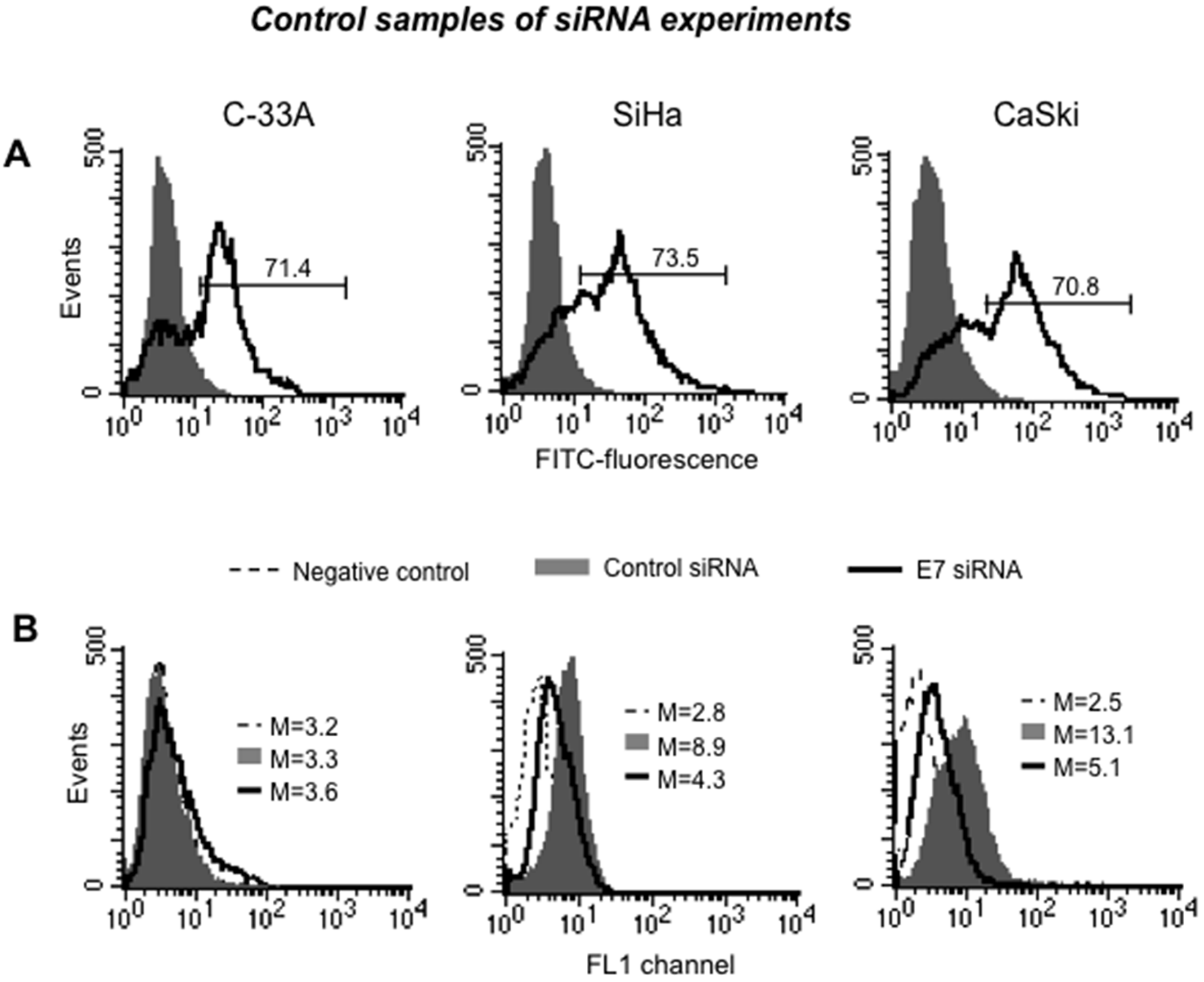

Supplementary Figure S3: siRNA experiment control samples. (**A**) Cytofluorimetric analysis of fluorescence emission in cells transfected with FITC-siRNA. The percentage of FITC-positive cells (corresponding to transfected cells) was considered indicative of the transfection efficiency. (**B**) Cytofluorimetric evaluation of E7 expression level 48 h after siRNA transfection. The numbers represent the median fluorescence intensity. A representative experiment among three is shown.

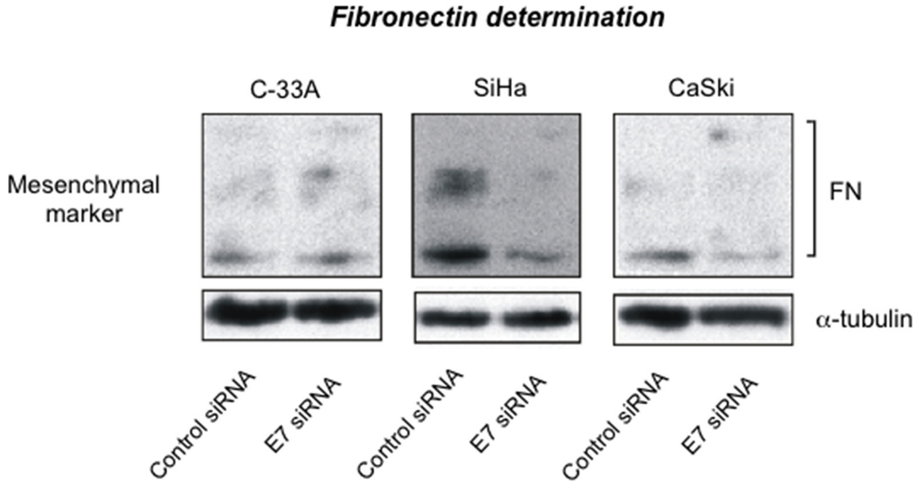

Supplementary Figure S4: Fibronectin determination in C-33A, SiHa and CaSki cells. Western blot analysis of fibronectin (FN) on C-33A, SiHa and CaSki cells transfected with control siRNA and E7 siRNA. FN appears as multiple bands, due to transcriptional and post-translational modifications (alternative splicing and glycosylation) of the protein. Loading control was evaluated using an anti-α-tubulin MAb.
